# Appendectomy history is associated with severe disease and colchicine resistance in adult familial Mediterranean fever patients

**DOI:** 10.3906/sag-2011-74

**Published:** 2021-08-30

**Authors:** Erdal BODAKÇİ, Nazife Şule YAŞAR BİLGE, Nuh ATAŞ, Berkan ARMAĞAN, Hasan SATIŞ, Alper SARI, Reyhan BİLİCİ SALMAN, Gözde KÜBRA YARDIMCI, Hakan BABAOĞLU, Levent KILIÇ, Mehmet AKİF ÖZTÜRK, Berna GÖKER, Şeminur HAZNEDAROĞLU, Umut KALYONCU, Abdurrahman TUFAN, Timuçin KAŞİFOĞLU

**Affiliations:** 1 Department of Internal Medicine, Faculty of Medicine, Osmangazi University, Eskişehir Turkey; 2 Department of Internal Medicine, Faculty of Medicine, Gazi University, Ankara Turkey; 3 Department of Internal Medicine, Faculty of Medicine, Hacettepe University, Ankara Turkey

**Keywords:** Familial Mediterranean fever, appendectomy, surgery, prognosis, colchicine resistance

## Abstract

**Background/aim:**

Peritonitis attacks of Familial Mediterranean Fever (FMF) usually requires emergency medical admissions and it’s hard to distinguish a typical abdominal attack from surgical causes of acute abdomen. Therefore, history of abdominal surgery, particularly appendectomy, is very common in patients with FMF. However, history of appendectomy might also give some clues about the course of FMF in the adulthood. This study was to determine whether the history of appendectomy help to anticipate disease course of FMF in the adulthood.

**Materials and methods:**

All patients recruited from FMF in Central Anatolia (FiCA) cohort, comprising 971 adult subjects. All patients fulfilled the Tel Hashomer criteria. Demographic data, FMF disease characteristics, co-morbid conditions, past medical history, surgical history and disease complications were meticulously questioned and laboratory features and genotype data (if available) were recruited from patient files.

**Results:**

Appendectomy history was evident in 240 (24.7%) subjects. Disease onset was earlier and peritonitis is strikingly more prevalent (97.1% vs. 89.6%, p < 0.001) in appendectomized patients. These patients had reported almost two fold more frequent attacks in the last year compared to appendix intact patients (median 3.5 vs. 2 attacks, p = 0.001) without a difference in frequency of musculoskeletal and skin attacks. Severe disease was more common (10% vs. 5.9%, p = 0.038) due to involvement of more attack sites throughout the life and more frequent attacks. Appendectomy patients had used higher daily doses of colchicine to control disease (1.43 ± 0.6 mg vs. 1.27 ± 0.52 mg, p = 0.002) but colchicine resistance was also more common in these patients, 15% vs. 6.7% respectively, p < 0.001.

**Conclusion:**

Appendectomy history is common in FMF patients and associated with frequent serositis attacks in adulthood. These patients require higher colchicine doses with a lower rate of response and more need for Interleukin-1 antagonist therapies.

## 1. Introduction

Familial Mediterranean fever (FMF) is the most common hereditary autoinflammatory disorder characterized by recurrent attacks of fever, peritonitis, pleuritis, synovitis, and erysipelas-like erythema [1,2]. It frequently occurs among Turks, Armenians, Jews, and Arabs, but several hundreds of patients described in non-Mediterranean countries [3]. Disease onset is prior to 10 years of age in 60% of cases and before age 20 in 90% of cases, although disease may start at any age [4]. Typical FMF attacks are self-limited and last between 1 to 3 days without a sequela. There is no diagnostic laboratory marker and genetic analysis by itself has low sensitivity and specificity; hence, the diagnosis of FMF relies mainly on clinical criteria [5]. Due to rarity of disease and lack of specific diagnostic tests most cases of FMF remain undiagnosed or misdiagnosed [6]. 

The most common manifestation of FMF is episodic abdominal pain resulting from sterile peritonitis which occur 95 percent of the patients [4]. Abdominal pain and tenderness usually start as localized and then progress to become more generalized. Since the cause of abdominal pain is the inflammation of the peritoneum, signs of peritonitis such as guarding, rebound tenderness, rigidity, and adynamic ileus are often present, a clinical emergency scenario quite similar to “acute surgical abdomen”. The most common cause of acute surgical abdomen in childhood is acute appendicitis with a peak incidence between 10–19 years. The lifetime risk of acute appendicitis is 7%–9%, which is far beyond the prevalence of FMF [7] which has a prevalence of 1/400–1/1000 in endemic countries [8]. 

In clinical practice, appendicitis, ileus/subileus, cholecystitis, and renal colic may be perceived as causes of abdominal pain before a reliable diagnosis of FMF has been made, which may lead to misdiagnosis and inappropriate treatments [9]. Heterogeneity of FMF attacks, in terms of severity and duration, and their erratic recurrence and remission, together with the rare incidence and low awareness, explain diagnostic delays of these disorders and the high rate of unnecessary laparotomies and appendectomies [9]. Therefore, routine preoperative screening for a personal or family history of symptoms compatible with FMF, should undoubtedly reduce the frequency of unwarranted surgical interventions. 

In patients with FMF, appendectomy is usually performed in childhood prior to the diagnosis, however even confirmation of FMF diagnosis, some patients underwent laparotomy due to difficulties in distinguishing FMF peritonitis from acute surgical abdomen causes. In previous reports abdominal surgery history rate is as high as 20% in FMF patients [10]. Our observation is that, patients with history of appendectomy or laparotomy experience more attacks than those who did not have these operations which may give a clue about the clinical course of FMF in adulthood. Herein, we aimed to investigate clinical features and disease course of FMF patients with history of appendectomy in a large cohort.

## 2. Materials and methods

In this study, we enrolled 971 adult patients with FMF who followed at the outpatient rheumatology clinics of three university hospitals: Gazi University, Hacettepe University, and Eskişehir Osmangazi University Hospitals which admit patients from all over the country. The study was conducted between January 2018 and December 2018. The diagnosis of FMF was made according to the Tel Hashomer criteria [11]. This study was approved by Gazi University ethical committee (approval no. 2017-622), conducted in accordance with the 1975 Helsinki Declaration. Informed consent was obtained from all participants before the inclusion of study.

 Demographic data, FMF disease characteristics, co-morbid conditions, past medical history including surgeries, medication history, colchicine doses, and disease complications were meticulously questioned and laboratory features were recruited from patient files. Disease severity and FMF associated damage were assessed with International Severity Scoring System for FMF (ISSF) and Autoinflammatory Disease Damage Index (ADDI), respectively [12,13]. Having less than one attack per 6-months-period is defined as complete response to colchicine. Colchicine resistance (crFMF) was defined as experiencing more than one attack per month despite the maximal tolerated dose of colchicine [14].

In ADDI, damage is defined as “persistent or irreversible change in structure or function, that is present for more than 6 months” [12]. ADDI contains 18 items, and these items are categorized by organ systems as follows: reproductive, renal/amyloidosis, developmental, serosal, neurological, ears, ocular, and musculoskeletal. The renal/amyloidosis and neurological damage categories were assigned to have the highest number of points while serosal damage got the lowest. This index provides a universal instrument to measure damage by chronic inflammation in FMF [15].

ISSF was developed to measure disease severity in children and adult FMF patients for research purposes and clinical practice. It consists of nine clinical and laboratory variables: chronic sequela, organ dysfunction, organ failure, attack frequency, increased acute-phase reactants, involvement of more than two sites during a single episode, more than two different types of attack during the course of the disease, duration of attacks, and exertional leg pain [13]. The maximum score is 10 and the degree of severity was determined as mild (≤2), intermediate (3–5) or severe disease (≥6) [12]. 

For statistical analyzes, patients were divided into two groups according to their history of appendectomy as Appendectomy Performed (AP) or Appendix Intact (AI). Differences between attack features, disease severities, damage accrual and treatment responses were compared. Categorical variables were given as numbers and percentages, and continuous variables were presented with their mean ± standard deviation (SD) or median (25%–75% interquartile ranges) for descriptive analysis. The conformity of continuous variables to normal distribution was evaluated by using visual (histogram and probability graphs) and analytical methods (Kolmogorov–Smirnov/Shapiro–Wilk tests). If variables were normally distributed Student-t-test was used, if not Mann–Whitney U test was used for comparison of data sets. The chi-square test was used for the comparison of categorical variables. p < 0.05 was considered as statistically significant in all analyzes. 

## 3. Results

The study comprised of 971 FMF patients (597 female, mean age 35.3 ± 12.1 years). Appendectomy history was evident in 240 (24.7%) subjects. AP group is a bit older and had an earlier disease onset (Table 1). In AP group mutation negative patients were less and those with two mutations were more common, without a difference in M694V homozygous mutation. Considering types of attacks evaluated as ever experienced till inclusion, peritonitis is strikingly more prevalent in AP group (97.1% vs. 89.6%, p < 0.001). Arthritis, skin rash and myositis were also more common in AP group compared to AI group (Table 1). 

**Table 1:** Demographic, clinical, and attack features of patients Familial Mediterranean fever.

			Appendectomy performed (AP) group (n = 240)	Appendix intact (AI)group (n = 731)	p
Age, years*			37.2 ± 11.8	34.6 ± 12.1	0.004
Female			151 (62.9)	446 (61.0)	0.6
Male			89 (37.1)	285 (38.9)	
FMF onset ≤10 years old			137 (57.1)	357 (48.8)	0.027
Age at FMF diagnosis, years*			24.8 ± 12.9	24.4±13.2	0.7
Number of mutations		Mutation negative	14 (5.8)	60 (8.2)	0.009
		Single mutation	58 (24.2)	256 (35.0)	
		Double mutation	118 (49.2)	308 (42.2)	
Mutation unknown			50 (20.8)	107 (14.6)	0.28
Harboring M694V mutation			150 (65.5)	469 (64.2)	0.15
M694V homozygous			60 (25)	165 (21.7)	0.17
Fever			206 (85.8)	601 (82.2)	0.22
Peritonitis			233 (97.1)	655 (89.6)	<0.001
Pleuritis			120 (50)	345 (47.2)	0.43
Arthritis			117 (48.7)	303 (41.4)	0.043
Erysipelas like erythema			78 (32.5)	176 (24.1)	0.009
Myalgia			64 (26.7)	151 (20.6)	0.053
Family history of amyloidosis			14 (6.2)**	31 (5.3)***	0.6
Disease severity (ISSF score)*			3.13 ± 1.67	2.73 ± 1.48	0.001
ISSF	Mild disease		102 (42.5)	361 (49.4)	0.038
	Intermediate disease		114 (47.5)	327 (44.7)	
	Severe disease		24 (10)	43 (5.9)	

All data are presented as number (%), or *mean ± standard deviation. Data available for **226 and ***587 patients, respectively. FMF: familial Mediterranean fever, ISSF: International Severity Scoring System.

AP group had reported almost twice as many attacks compared to AI group, in the last year (median 3.5 vs. 2 attack, p = 0.001), of the study inclusion. When attacks were individually analyzed, there were differences in the frequency of fever, peritonitis and pleuritis attacks in the last year (Table 2). However, there was no difference in the frequency of musculoskeletal and skin attacks between AP and AI groups. Severe disease, assessed with ISSF, is more common in AP group (10% vs. 5.9%, p = 0.038) due to involvement of more disease sites and more frequent attacks. 

**Table 2:** Disease course and prognostic parameters of Familial Mediterranean fever patient groups in the last year before enrollment.

	Appendectomy performed (AP)group/median (n = 240)	Appendix intact (AI)group/median (n = 731)	p
Number of all type of attacks in the last year	3.5 (0–10)	2 (0–5)	0.001
Fever	2 (0–5)	1 (0–3)	0.005
Peritonitis	2 (0–6)	1 (0–4)	0.014
Pleuritis	2.5 (0–5)	1 (0–4)	0.03
Arthritis	1 (0–4)	1 (0–3)	0.4
ELE	0 (0–3)	0 (0–2)	0.9
Myalgia	3 (0–10)	2 (0–5)	0.1
Colchicine dose in the last visit, mg*	1.43 ± 0.60	1.27 ± 0.52	0.002
Colchicine nonresponse, n (%)	36 (15.0)	49 (6.7)	<0.001
Anti-IL1 antagonist use, n (%)	31 (12.9)	59 (8.1)	0.025
Chronic inflammation, n (%)	37 (15.4)	105 (14.4)	0.6
Median CRP in the last visit, mg/L	11 (6–18)	12 (6–26)	0.26
Amyloidosis, n (%)	14 (5.8)	43 (5.9)	0.97
Damage accrual (ADDI score)	1 (0–1)	1 (0–1)	0.24
Total number of abdominal surgeries till inclusion of study	2 (0–2)	0 (0)	<0.001

Data are presented as median (interquartile range 25–75 percentile), *mean ± standard deviation or number (%). ADDI: autoinflammatory disease damage index, CRP: C reactive protein, ELE: erysipelas like erythema, IL-1: interleukin-1.

AP group had used higher doses of colchicine to control their disease (1.43 ± 0.6 mg vs. 1.27 ± 0.52 mg, p = 0.002). Colchicine resistance was also more prevalent in AP group, 15% vs. 6.7% respectively, p < 0.001. The proportion of patients in AP group is higher for anti-IL-1 antagonist (anakinra or canakinumab) use. History of other abdominal surgeries such as, cholecystectomy or gynecologic operations were also more common in AP group (median total number of abdominal surgery 2 vs. 0 in AP and AI groups respectively, <0.001). Although severe disease was more common in AP group, no difference in FMF associated complications was observed, such as prevalence of chronic inflammation, amyloidosis and damage accrual. 

## 4. Discussion 

Results of this study shows that appendectomy history is extremely common in FMF patients. Moreover, patients with history of appendectomy had reported more frequent abdominal operations. Having history of appendectomy gives some clues about the course of FMF in adulthood. These patients report more frequent attacks particularly serositis type episodes despite they use higher doses of colchicine. Colchicine nonresponse rate is higher and the need for anti-IL-1 therapies are more pronounced in appendectomized patients. 

FMF patients most frequently present initially with acute abdominal pain and fever, both of which are also the common causes of emergency department admissions. Emergency department is the most common medical care unit for the first time admissions of FMF patients. As many patients with acute abdominal pain are discharged from the emergency department without a definitive diagnosis, it is not surprising that, due to its rarity, most cases of FMF remain undiagnosed or are misdiagnosed as acute appendicitis or other causes of acute surgical abdomen [6]. The diagnosis of FMF requires a high index of suspicion and careful assessment of clinical history. Family history, clinical features, in particular history of previous self-limiting acute attacks, together with prodromal symptoms and triggering factors, are useful to suspect FMF but all of them are seldom questioned in emergency department admissions [6]. 

In our study, it was observed that there is a diagnostic delay of 10–12 years between the onset of FMF complaints and the age of diagnosis. Childhood is the peak period for the diagnosis of acute appendicitis and disease onset before the age of 10 is more common in appendectomized patients in our study. It is probable that appendectomy was performed with a diagnosis of acute appendicitis in the first or second attack before the FMF diagnosis was made. Due to difficulties in distinguishing acute appendicitis, lack of previous diagnosis of FMF and hazy clinical picture in children might apt to surgeons to operate. Additionally, longer disease duration might increase emergency admissions which might increase risk of appendectomy. Furthermore, in some patients despite an established diagnosis of FMF, appendectomy is still performed because of problems in distinguishing typical attack from appendicitis. According to a study conducted in Israel, the frequency of appendectomy in FMF patients was reported to be 16% [16] and in a nationwide multicenter study evaluating 2838 Turkish FMF patients was 19% in 2005 [10]. This rate was 24.7% in our study, which might be lack of knowledge on FMF and pathogenesis of autoinflammatory diseases among physicians as well as low public awareness of FMF in the past decades. 

Colchicine is the mainstay of FMF treatment. It is generally safe and well-tolerated. Efficacy of colchicine has been shown in reducing typical attacks and prevention of secondary amyloidosis. The recommended dose of colchicine is 1–2 mg/day for children older than 10 years and for adults. However, some patients cannot tolerate effective doses of colchicine due to its side effects. The most common side effects of colchicine are diarrhea, abdominal discomfort and nausea [17,18]. In addition, despite regular use of highest therapeutic doses, about 10% of patients do not respond sufficiently to the colchicine [14]. Overall, approximately 60% of patients respond well to daily colchicine treatment, while 20%–30% show partial response and 5%–10% have no response [19]. Similar rate was found in our study with being 8.7% of patient’s colchicine resistant. However, the rate of colchicine resistance was twice as more in AP group (15% vs. 6.7%) despite they had used higher doses of colchicine. Therefore, history of appendectomy might be instructive in terms of treatment planning, such as colchicine dosages, estimation of colchicine response and the need for anti-IL-1 treatments.

In our study, ISSF score was 3.13 ± 1.68 in the AP group with more severe patients in appendectomy performed group. This is because of occurrence of more frequent attacks and involvement of more disease sites in AP group. However, when experienced attacks in the last year were analyzed which represent adulthood period, the difference was present for fever and peritonitis/pleuritis attacks, whereas, the number of musculoskeletal attacks and erysipelas like erythema in the last year were similar in both groups. Theoretically, appendectomy by itself might facilitate occurrence of serositis attacks as suggested by increased risk of inflammatory diseases among appendectomized patients in the long term such as inflammatory bowel disease and sepsis [20,21]. There was no difference between groups for the development of amyloidosis or damage accrual. Previous studies have shown that there is no correlation between frequency of attacks and the risk of amyloidosis, except for arthritis which significantly increases the risk of amyloidosis [22,23]. Frequency of arthritis attacks are the same in both groups in our study which may explain similar rate of amyloidosis and ADDI scores. 

Our study has some limitations. The most important limitation is the retrospective design of the study which impairs evaluation of pre-appendectomy FMF disease characteristics. The types, numbers and the duration of attacks were based on the declaration of patients which depends on patient recall ability. To overcome this, we included only the number and duration of previous attacks. The types of attacks were evaluated dichotomously as ever experienced or not. Another limitation is that we did not ask for pathologic examination of surgical specimens; therefore we cannot decide whether appendectomy or other operations performed upon true indication or not. But remarkably high prevalence strongly suggests unindicated surgeries. Finally, pain severity perceived by patients might be different and we did not evaluate pain severity, which is quite subjective with considering heterogeneity of attacks and lack of a standardized pain tool in FMF. 

In conclusion, appendectomy is common in FMF patients and careful history taking may reduce unnecessary operations. Appendectomy patients experience more frequent attacks, particularly fever, peritonitis/pleuritis attacks, they display more severe disease and poor response to colchicine. Hence, history of appendectomy might help to anticipate disease course and colchicine responses, as well as to make treatment plans.

## Author contributions

In accordance with ICMJE criteria, EB and TK, designed the study and wrote the initial draft of the manuscript. AT, UK, MAO, BG, NSYB, and SH contributed to the design of the study, the collection and interpretation of data, and the assistance of the preparation of the manuscript. BA, NA, HS, AS, NSYB, RBS, GKY, HB and LK contributed to the data collection and interpretation and revised the manuscript. The literature data were searched and analyzed by all authors. All authors approved the final version to be submitted for publication and agree to be accountable for all aspects of the work.

## Funding

This work was supported by grants from Hacettepe University Rheumatology Association (grant no: HRD2018-001).

## Ethical approval

This study was approved by Gazi University ethical committee (Approval no. 2017-622), conducted in accordance with the 1975 Helsinki Declaration. Informed consent Informed consent was obtained from all participants included in the study.
